# In Vitro Activity of Essential Oils from *Piper* Species (Piperaceae) against Tachyzoites of *Toxoplasma gondii*

**DOI:** 10.3390/metabo13010095

**Published:** 2023-01-06

**Authors:** Adalberto Alves Pereira Filho, Mariana Maciel Cunha, Mariana Alves Stanton, Lydia Fumiko Yamaguchi, Massuo Jorge Kato, Érica S. Martins-Duarte

**Affiliations:** 1 Departamento de Parasitologia, Instituto de Ciências Biológicas, Universidade Federal de Minas Gerais, Belo Horizonte 31270-901, Minas Gerais, Brazil; 2 Department of Fundamental Chemistry, Institute of Chemistry, University of São Paulo, São Paulo 05508-000, São Paulo, Brazil

**Keywords:** toxoplasmosis, essential oils, *Piper*

## Abstract

Toxoplasmosis is a tropical and neglected disease caused by the parasitic protozoa *Toxplasma gondii*. Conventional treatment with sulfadiazine and pyrimethamine plus folinic acid, has some drawbacks, such as inefficacy in the chronic phase, toxic side effects, and potential cases of resistance have been observed. In this study, the activity of essential oils (EOs) from three *Piper* species and their main constituents, including α-Pinene (*Piper lindbergii* and *P. cernuum*), β-Pinene (*P. cernuum*), and dillapiole (*P. aduncum*), were evaluated against tachyzoites of *T. gondii*. α-Pinene was more active [(IC_50_ 0.3265 (0.2958 to 0.3604) μg/mL)] against tachyzoites than *P. lindbergii* EO [0.8387 (0.6492 to 1.084) μg/mL]. Both α-Pinene and *P. lindbergii* EO exhibited low cytotoxicity against NHDF cells, with CC_50_ 41.37 (37.64 to 45.09) µg/mL and 83.80 (75.42 to 91.34) µg/mL, respectively, suggesting they could be of potential use against toxoplasmosis.

## 1. Introduction

*Toxoplasma gondii* (Nicolle and Manceaux, 1909) is an apicomplexan parasite present in approximately one-third of the human population worldwide. In addition to humans, it can infect practically all warm-blooded vertebrates. This zoonotic infection represents an important public health problem in human and veterinary medicine [1]. Transmission occurs mainly by ingestion of oocysts in the environment and of tissue cysts in raw or undercooked meat. Although most people affected are asymptomatic, serious cases can occur in congenitally infected newborns and in immunocompromised patients [2,3].

Toxoplasmosis treatment usually consists of a combination of sulfadiazine and pyrimethamine plus folinic acid. This combination has a synergic action and traditionally shows good results in the acute stage of infection [4,5]. However, these and the other currently recommended drugs for toxoplasmosis treatment have limitations. A notable limitation is that one of the mechanisms of action involves the reduction innucleic acid synthesis, which makes a teratogenic drug. Adverse effects, resistance, and intolerance against these and other known treatments are commonly reported in the literature. In addition, all drugs are inefficient against the chronic phase of infection. These limitations affect the success of the treatments, mainly in immunocompromised patients and in ocular and congenital cases, which raises the need for new treatment options [6,7,8,9,10,11].

In the search for alternatives to the treatment of toxoplasmosis, essential oils (EOs) can represent an excellent source of mixtures of biologically active natural products. In this context, the genus *Piper* L. (Piperaceae) is one of the most diverse and widely distributed plant groups in pantropical regions, with approximately 1000 species worldwide and several reported cases of use in traditional medicine. *Piper* species are aromatic plants and have many metabolites with demonstrated biological effects on human health, and therefore they represent a potential source of pharmacologically active substances.

In addition, EOs from *Piper* species have demonstrated antiparasitic activity against several medically important protozoa. Here, it is worth reporting some studies that have already been carried out against *Plasmodium falciparum* [12,13], *Trypanosoma cruzi* [12,13,14,15,16], and several species of the genus *Leishmania* [15,17]. Despite the absence of studies using EOs from the *Piper* genus against *T. gondii*, it is known that the ethanol extract from *Piper betle* L. resulted in 100% survival of infected mice with this apicomplexan parasite [18].

Due to the urgent need to find and develop new treatments for toxoplasmosis, the objective of the present work was to evaluate the potential of EOs from species of the genus *Piper* (Piperaceae) and some of their major compounds for use against *T. gondii*.

## 2. Materials and Methods

### 2.1. Parasites

The highly virulent *T. gondii* RH strain (Type I) was maintained in vitro through serial passages in 25 cm^2^ culture flasks of confluent neonatal normal human dermal fibroblast cells (NHDF (Lonza^®^, Catalog: CC-2509)) in complete RPMI medium (RPMI 1640 medium, Gibco, 2% fetal bovine serum, 4 mM of glutamine, 100 U/mL of penicillin, 100 μg/mL of streptomycin and 25 μg/mL of fungizone, Gibco). Culture flasks were maintained at 37 °C and 5% CO_2_.

### 2.2. Plant Materials and Extraction of Essential Oils 

The leaves of three species of plants belonging to the genus *Piper* with their respective identification vouchers in parentheses: *Piper aduncum* L. (K-0057), *Piper cernuum* Vell. (K-0137) and *Piper lindbergii* C.DC. (K-2325), were collected in the period of January to June 2018. The vouchers were identified by Dr. Eric Tepe and deposited at the Herbarium of USP, University of São Paulo for identification, and all collections were made under permits #59161-1 and 010/2018-R from SISBIO (Sistema de Autorização e Informação em Biodiversidade) and Fundação Serra do Japi, respectively.

The EOs were extracted from fresh leaves of each species, submitted to hydrodistillation in a Clevenger-type apparatus for 4 h, using 300–500 g of fresh leaves and 500 mL of distilled water [19,20]. The EOs were collected and dried with anhydrous sodium sulfate and stored in amber bottles in a refrigerator at 4 °C until the experiments and analysis by GC-MS and their main constituents were identified based on library search, retention index (RI), and use of standard compounds when available, and expressed as relative percentage of each constituent according to the method described [21]. 

The major compounds from EOs characterized by GC-MS and used in this study were: α-Pinene (Sigma-Aldrich: 147524) and β-Pinene (Sigma-Aldrich: 402753) and were acquired commercially. Pure dillapiole was obtained by fractionation using the Isolera Flash Chromatography system (Biotage INC) according to the method described [21]. 

### 2.3. Antiproliferative RH Strain Tachyzoites Delayed-Death Assays

**Figure 1:**
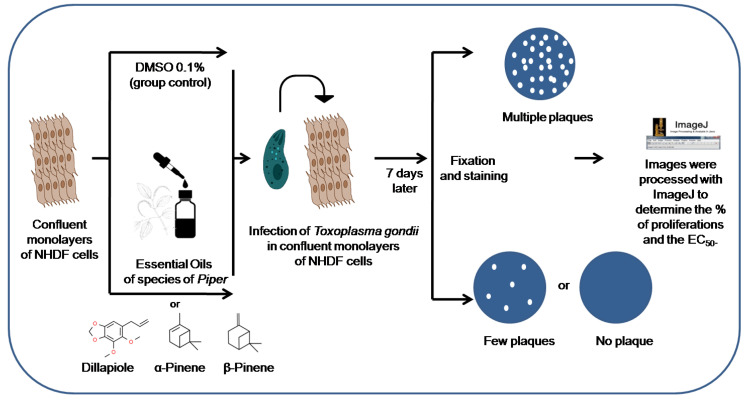
Overview of the assay used to evaluate the in vitro antiproliferative effect of *Piper* sp. essential oils and dillapiole, α-Pinene, and β-Pinene on *Toxoplasma gondii* tachyzoites of the RH lineage. The chemical structures of dillapiole, α-Pinene, and β-Pinene were obtained from the website ChemSpider.

NHDF cells were maintained in 6-well culture plates at 37 °C and 5% CO_2_. After verifying the formation of NHDF monolayers, the old medium was removed and a new complete RPMI-1640 medium was added, adding either EOs of *Piper* or the major compounds once, at final concentrations of 0.5, 1, 2.5, 5, 10, 15, 20 μg/mL. Subsequently, previously counted tachyzoites of the RH strain were added to achieve an amount of 800 tachyzoites/well. The plates were left for 7 days at 37 °C and 5% CO_2_. After verifying the infection of NHDF monolayers by *T. gondii* tachyzoites, the culture medium was discarded, and the plates were fixed with 99% ethanol and stained with crystal violet to visualize plaque formation. The plates were then photographed on an Alpha DigiDocphoto document. Images were processed with ImageJ software to determine plaque area. The areas of each well of the plate treated with EOs of *Piper* or major compounds were compared with the control treated only with 0.1% DMSO and the percentages of proliferations were calculated to obtain the respective 50% effective concentrations (EC_50_) (Figure 1). 

### 2.4. Invasion Assay of NHDF Cells by T. gondii Tachyzoites

Cultures of the RH strain that had completely lysed an NHDF monolayer were recollected into a centrifuge tube. Additionally, 1 × 10^6^ extracellular tachyzoites resuspended in RPMI medium were preincubated for 2 h at 37 °C and 5% CO_2_ based on the EC_50_ of each compound or of the vehicle (DMSO 0.1%). Parasites were centrifuged at 6000 rpm × 5 min three times, resuspended in RPMI medium, and then allowed to invade NHDF cell monolayers previously grown on coverslips in 24-well plates by incubation for 2 h at 37 °C. The cultures were then washed to remove extracellular parasites and fixed with Bouin for 20 min, washed again with 70% alcohol, and stained with solutions 2 and 3 of the Fast Panoptic kit (Laborclin) for 45 and 10 s, respectively. The glass slides were observed under the optical microscope, cells were counted, and the percentage of infected cells was calculated from the ratio of infected cells to uninfected cells.

### 2.5. Host Cell Viability

The viability of NHDF cells was evaluated by MTS/PMS colorimetric assay. The cells were treated once with *Piper* EOs or their major compounds (dillapiole, α-Pinene, and β-Pinene) at final concentrations of 12.5, 25, 50, 100, and 200 μg/mL. For that, fibroblasts were seeded in 96-well plates at a density of 1 × 10^5^ cells/well. After semi-confluence, cells were incubated with complete RPMI medium in the absence or presence of EOs or of single compounds for 7 days at 37 °C and 5% CO_2_. At the end of the incubation time, the cells were washed with sterile PBS (pH 7.2), and the wells filled with 100 μL of PBS + 10 mM of glucose and 20 μL of the MTS/PMS reagent (20:1), from a stock solution of 2 mg/mL MTS and 0.92 mg/mL PMS diluted in PBS (Promega, Madison WI, USA). After 2 h of incubation, the absorbance of the samples was detected at 490 nm using the Versamax ELISA plate reader (Molecular Devices) and cytotoxicity was calculated as the percentage of viable cells in relation to control cells. The cytotoxic concentration of 50% (CC_50_) for the host cells was determined, and the selective index (SI) was calculated as the ratio of CC_50_/EC_50_ [22].

### 2.6. Statistical Analysis

The data were organized in spreadsheets using Microsoft Excel software (Office 2007). The obtained data were subjected to non-linear regression to obtain 50% effective concentrations (EC_50_) and cytotoxic concentration (CC_50_), with 95% confidence intervals using the GraphPad Prism 7.0 software (GraphPad Inc., San Diego, CA, USA). Comparisons between groups (control group and treated groups) were carried out using Kruskal–Wallis followed by Dunn’s posthoc test. Statistical significance was defined as *p* < 0.05.

## 3. Results

### 3.1. Chemical Composition of Essential Oils (EO)

The chemical analysis of the EOs obtained from *Piper aduncum*, *P. cernuum* and *P. lindbergii* identified 34, 35, and 44 compounds, respectively, as shown in Table 1. In summary, the major compounds were identified as phenylpropanoids, sesquiterpenes, and monoterpenes. From the analysis by GC-MS, some major compounds of the EOs were selected to be used in the tests: α-Pinene, β-Pinene, and Dillapiole.

### 3.2. In Vitro Antiproliferative Effect of Piper EOs and Major Compounds on RH Strain Tachyzoites

**Figure 2:**
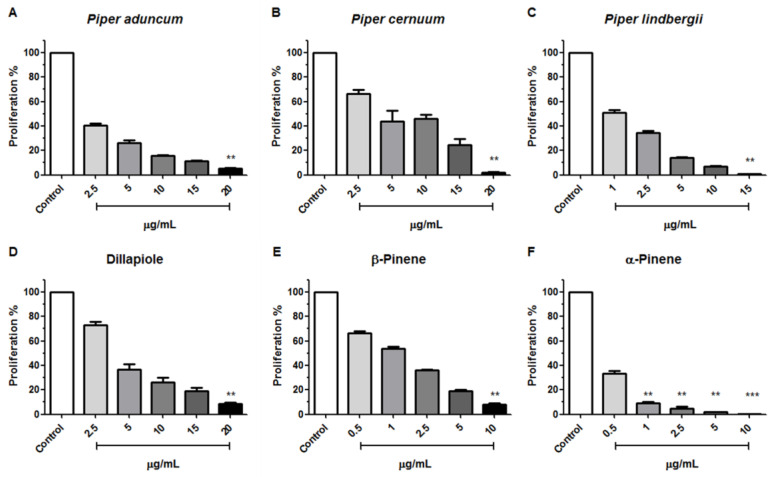
Antiproliferative effects of essential oils from three *Piper* species (**A**–**C**) and dillapiole, β-Pinene and α-Pinene (**D**–**F**) on tachyzoites of the *T. gondii* RH strain 7 days after treatment. Kruskal–Wallisfollowed by Dunn’s posthoc test was performed (** *p* < 0.001, and *** *p* < 0.0001 among the compared groups).

The antiproliferative effect of *Piper* EOs and three major compounds against tachyzoites of the *T. gondii* RH strain were evaluated after 7 days of treatment under the same conditions as described above in Section 2.3. All compounds and EOs evaluated were able to inhibit the proliferation of *T. gondii* and had their effective concentrations calculated (Table 2). The EO from *P. lindbergii* was the most active mixture and inhibited the proliferation of *T. gondii* with an EC_50_ = 0.839 µg/mL, in contrast to the *P. cernuum* EO, which presented the highest EC_50_ = 3.687 µg/mL. As for the major components, α-Pinene showed the lowest EC_50_ = 0.326 µg/mL, followed by β-Pinene EC_50_ = 1.145 µg/mL, and finally dillapiole with EC_50_= 4.827 µg/mL. Excepting *P. cernuum* EO, all tested compounds and EOs were able to inhibit *T. gondii* proliferation in a dose-dependent manner (Figure 2A–F).

### 3.3. Cytotoxicity Evaluation of Piper EOs and Major Compounds in NHDF Cells

**Figure 3:**
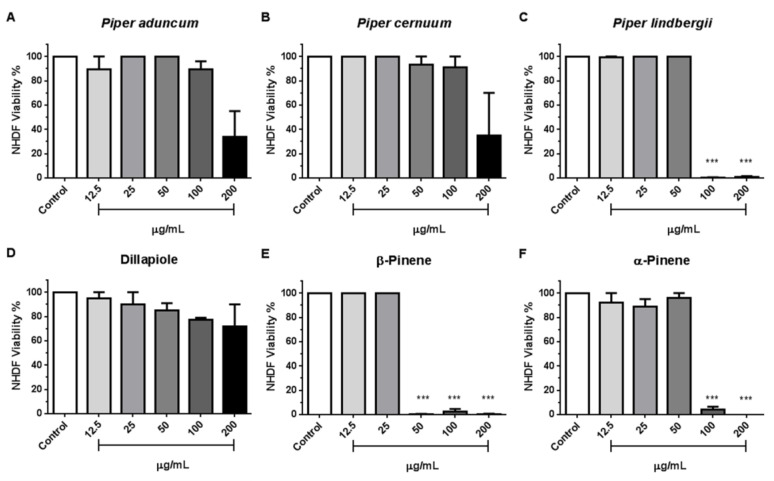
Cytotoxic effects of *Piper* sp. essential oils (**A**–**C**) and dillapiole, β-Pinene, and α-Pinene (**D**–**F**) on NHDF cells. Viability was compared by Kruskal–Wallis, followed by Dunn’s posthoc test (*** *p* < 0.0001 among the compared groups).

To verify whether *Piper* EOs and major compounds inhibit *T. gondii* proliferation without exerting a secondary toxic effect against the host cell, NHDF monolayers were treated with different concentrations (12.5–220 µg/mL) for 7 days under the same conditions as in Section 2.5 above. The selectivity index (SI) showed that *Piper* EOs and major compounds have a wide selectivity index ranging from 46 to 126 (Table 3). *P. lindbergii* and α-Pinene showed the highest levels of selectivity, 99 and 126 SI, respectively. Except for Dillapiole, which affected the cytotoxicity in a dose-dependent manner, all the other compounds and EOs showed cytotoxicity variation that did not follow a linear pattern (Figure 3A–F).

### 3.4. Evaluation of the Effect of Major Compounds on the Invasion of NHDF Cells by T. gondii Tachyzoites of the RH Strain

**Figure 4:**
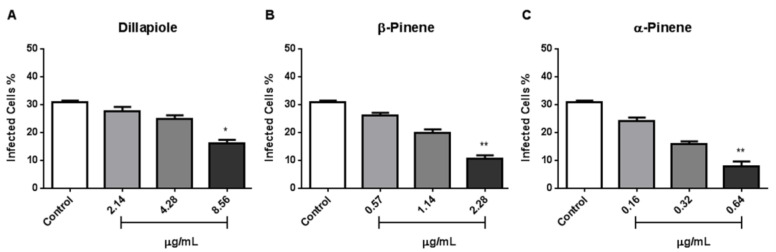
Effect of pretreatment with Dillapiole, β-Pinene, and α-Pinene (**A**–**C**) on *T. gondii* tachyzoites during NHDF cell invasion. The number of infected cells in each group was compared to controls by Kruskal–Wallis, followed by Dunn’s posthoc tests (* *p* < 0.01, and ** *p* < 0.001 among the compared groups).

To verify whether single major compounds showed influence on the invasion of NHDF cells by *T. gondii*, 10^6^ tachyzoites were pre-treated under the same conditions as in Section 2.4 above. Invasion assays were also performed to evaluate the behavior of *T. gondii* after the pre-treatment with the major compounds tested here, to verify if they could harm this important stage of the parasite’s cycle. Treatment with α-Pinene led to the lowest rates of invasion, with averages of 5.5% at the highest concentration tested. In contrast, dillapiole showed low effects on *T. gondii* invasion of NHDF cells (Figure 4).

## 4. Discussion

Studies focusing on natural products and their anti-*T.gondii* effects represent alternatives for the treatment of toxoplasmosis. Herein, we describe the in vitro activity of the EOs from three species of genus *Piper* and their main constituents: α-Pinene, β-Pinene, and Dillapiole against tachyzoites of *T. gondii*.

In general, the ability of species of the genus *Piper* to show activity against parasites of medical importance is well known. However, some works need to be highlighted. A classic example is the EO from *Piper aduncum* that showed activity against *Plasmodium falciparum*, *Trypanosoma cruzi*, *Trypanosoma brucei*, and *Leishmania infantum* [23].

Several studies have demonstrated the antiparasitic effects of the genus *Piper* on medically important protozoa, but on *Toxoplasma gondii* there are only reports of the use of ethanolextracts from *P. betle*, *P. nigrum* and *P. sarmentosum* [18]. Our study revealed that EOs of *Piper* species and some of their constituents were active against *T. gondii*.

Analysis of the chemical composition of the EOs of three *Piper* species (Table 1) and comparisons to the anti-*T. gondii* activities suggest that, in the case of the *P. lindbergii* EO, the monoterpene α-Pinene could account for this potential. That is evidenced by the expressive amount of α-Pinene found in *P. lindbergii* (61.67%), compatible with their lowest EC_50_ values (Table 2). On the other hand, although *P. aduncum* contained 81.01% of Dillapiole in its composition, it had the second lowest EC_50_ among the three *Piper* species tested. Interestingly, isolated Dillapiole had the lowest EC_50_ suggesting other minor compounds in *P. aduncum* EO could account for the high activities against this parasite. Finally, although *P. cernuum* EO has both β-Pinene and α-Pinene (which showed *anti- T. gondii* activity), it performed worse than *P. aduncum* EO against *T. gondii*. That also suggests that some other compound in the *P. cernuum* may have a negative effect on the activity of this oil against *T. gondii*. EOs are complex mixtures of monoterpenes, sesquiterpenes, and phenylpropanoids, and the main components can act synergistically or antagonistically with minor constituents to modulate each other’s activity; consequently, we found different results than would be expected for single compounds [24].

*Toxoplasma gondii* is an obligate intracellular protozoan, and the host cell invasion process is crucial for parasite viability and infection establishment [25,26]. Thus, we sought to understand whether the pre-treatment of *T. gondii* tachyzoites with major compounds would be able to interfere with this essential process for the success of the parasitic infection. The pre-treatment of tachyzoites for 2 h with the major compounds of *Piper* EOs showed a decrease in the percentage of invading parasites, especially for β-Pinene and α-Pinene treatments. Similarly, a study using oleoresins from *Copaifera reticulata*, *C. duckei*, and *C. pubiflora* on *T. gondii* tachyzoites for one hour (with 64 and 32 μg/mL) significantly reduced parasite invasion compared to the control group [27].

In studies involving the search for new potential drugs for the treatment of toxoplasmosis, it is important to investigate their cytotoxicity given that the parasite is an obligatory intracellular organism. Moreover, in vitro toxicity tests are necessary for the initial phase of the discovery of promising drugs, as they constitute an essential tool to exclude compounds with cytotoxic properties [28,29]. The higher the value of the SI selectivity index obtained, the less toxic to the host and more selective to the parasite the compound is [30]. The selectivity indexes found in this work were highly satisfactory (99 for *P. lindbergii* and 126 for α-Pinene) since the selectivity index between parasites and host cells must reach at least 20 [31,32]. The selectivity potential of the EO of the genus *Piper* is already well known since previous works have demonstrated such action, either with the EO of *P. aduncum* with a high index of selectivity for trypomastigotes of *Trypanosoma cruzi* [12] or even against the promastigotes of *Leishmania major*, *L. mexicana*, *L. braziliensis* and *L. donovani* with a favorable selectivity index against peritoneal macrophages from BALB/c mice [33].

The present work opens future perspectives to explore the *Piper* EOs in the search for new compounds against toxoplasmosis and highlights that complex mixtures need to be further studied to understand the effects and interactions between their constituents on biological activity. In addition, it highlights α-Pinene as a promising compound for in vivo testing and against *T. gondii* bradyzoites.

## 5. Conclusions

The EO from *P. lindbergii* and its major monoterpene α-Pinene showed excellent anti-*T. gondii* activity, displaying a negative influence on the invasion of the parasite in the studied model, with a good selectivity index. These findings support future studies with these compounds using in vivo models of activity against *T. gondii* to search for new compounds and targets for the development of alternatives for the treatment of toxoplasmosis.

## Figures and Tables

**Table 1:** Chemical composition of the essential oils of three *Piper* species.

Compounds	RI^a^	RI^b^	PAD	PLD	PCE
α-Pinene ^S^	932	932	0.2	61.7	16.6
Camphene ^S^	947	946	-	1.7	0.1
β-Pinene ^S^	975	974	0.3	1.4	11.5
β-Myrcene ^S^	992	988	-	0.3	1.0
α-Phellandrene ^S^	1004	1002	0.1	-	0.2
2-Carene ^S^	1010	1008	0.1	-	0.2
α-Terpinene	1016	1014	-	-	4.5
p-Cymene ^S^	1024	1020	0.1	1.0	9.2
Limonene ^S^	1039	1024	0.1	5.3	0.8
(*Z*)-β-Ocimene ^S^	1039	1032	1.6	-	0.1
(*E*)-β-Ocimene ^S^	1049	1044	3.4	-	0.3
γ-Terpinene	1059	1054	0.2	-	9.9
α-Terpinolene ^S^	1088	1086	0.4	-	2.7
Linalool ^S^	1100	1095	-	1.6	-
(*E*)-4,8-Dimethyl-1,3,7-nonatriene (DMNT) ^S^	1117	1114	-	-	0.3
Camphor ^S^	1144	1141	-	1.1	-
Terpinen-4-ol	1178	1174	-	0.1	0.3
α-Terpineole	1191	1186	-	2.3	0.3
Oxygenated monoterpene *	1209	-	0.1	0.1	-
(+)-Piperitone	1255	1249	0.7	-	-
δ-Elemene	1339	1335	0.1	1.1	0.4
α-Cubebene	1352	1345	-	0.1	0.3
α-Ylangene	1374	1373	0.1	-	-
α-Copaene ^S^	1378	1374	0.2	6.4	2.1
β-Bourbonene	1387	1387	-	0.2	0.6
β-Elemene	1394	1389	0.2	0.3	4.4
α-Gurjunene ^S^	1412	1409	0.1	-	0.1
(*E*)-β-Caryophyllene ^S^	1422	1417	0.8	0.5	7.0
β-Gurjunene	1432	1431	0.2	0.6	0.5
(+)-Aromadendrene ^S^	1442	1439	-	0.2	0.4
α-Humulene ^S^	1457	1452	0.9	0.2	2.1
(-)-Alloaromadendrene ^S^	1464	1458	-	0.9	0.1
Dehydro-aromadendrane	1466	1460	-	0.9	-
γ-Muurolene	1480	1479	-	1.3	0.5
Germacrene D	1484	1481	2.7	-	5.2
β-Selinene	1490	1490	-	0.3	0.5
α-Selinene	1500	1498	1.4	0.1	-
Bicyclogermacrene	1500	1500	2.3	-	10.7
α-Muurolene	1503	1500	0.1	1.0	-
α-Bulnesene	1510	1509	0.2	-	0.7
γ-Cadinene	1517	1513	0.1	1.2	0.4
δ-Cadinene	1522	1522	1.2	-	-
(*E*)-Cadina-1.4-diene	1527	1533	-	1.3	0.3
Germacrene B	1561	1559	0.2	-	0.1
(*E*)-Nerolidol ^S^	1566	1561	0.1	-	0.9
Palustrol	1572	1567	-	-	0.1
Spathulenol	1581	1577	0.1	0.3	0.7
(-)-Caryophyllene oxide ^S^	1587	1582	-	3.4	0.8
Veridiflorol	1596	1592	0.3	0.5	0.6
β-Asarone	1623	1616	-	0.3	-
Dillapiole ^S^	1632	1620	81.0	-	0.1
epi-α-Muurolol	1646	1640	0.3	0.6	0.6
Torreyol	1650	1644	-	0.3	0.4
α-Cadinol	1659	1652	-	0.9	1.2
Apiole ^S^	1686	1677	0.2	-	-

RI^a^—Retention Index calculated against RI^a^. RI^a^: Retention index calculated against C8–C40 n-alkanes using an HP-5 ms column. RI^b^: Retention index values from the literature (Adams, 2007). PAD: *Piper aduncum*; PLD: *Piper lindbergii*; PCE: *Piper cernuum*. ^S^ Compound identity confirmed with an authentic standard. The remaining compounds were identified by comparing the RI and mass spectra with the Adams and Wiley databases (see text for details). * Unidentified compound.

**Table 2:** Evaluation of effective concentrations (EC_50_) of *Piper* sp. Essential oils (EOs) and major compounds on tachyzoites of the *T. gondii* RH strain.

EO/Compound	EC_50_ (95% Confidence Intervals) µg/mL	R
*Piper aduncum*	1.749 (1.498 to 2.043)	0.9807
*P. cernuum*	3.687 (2.190 to 6.208)	0.7144
*P. lindbergii*	0.839 (0.6492 to 1.084)	0.9358
Dillapiole	4.287 (3.511 to 5.234)	0.9331
β-Pinene	1.145 (1.008 to 1.300)	0.9836
α-Pinene	0.326 (0.295 to 0.360)	0.9805

**Table 3:** Evaluation of cytotoxicity concentrations (CC_50_) of *Piper* sp. Essential oils (EOs) and major compounds in NHDF cells. SI = selectivity index.

EO/Compound	CC_50_ (95% Confidence Intervals) µg/mL	R	SI
*Piper aduncum*	169.8 (135.6 to 212.8)	0.8074	97
*Piper cernuum*	172.1 (125.6 to 235.7)	0.6826	46
*Piper lindbergii*	83.80 (75.42 to 91.34)	0.9998	99
Dillapiole	210.8 (194.6 to 228.3)	0.9906	49
β-Pinene	70.78 (53.22 to 94.12)	0.9682	61
α-Pinene	41.37 (37.64 to 45.09)	0.9991	126

## Data Availability

Data areavailable in the manuscript.
